# Long-Acting Injectable Versus Oral Antipsychotics in Early Psychosis: An Umbrella Review of Systematic Reviews and Meta-Analyses

**DOI:** 10.1192/bjo.2026.11063

**Published:** 2026-06-30

**Authors:** Oluwatobi Idowu, Nicholas Aderinto, Temitomi Jane Oyedele

**Affiliations:** 1 Cheshire and Wirral Partnership NHS Foundation Trust, Chester, United Kingdom; 2 Ladoke Akintola University of Technology, Ogbomoso, Nigeria; 3 Bowen University, Iwo, Nigeria

## Abstract

**Aims:**

Long-acting injectable antipsychotics (LAIs) are commonly used alternatives to oral antipsychotics (OAPs) in treating schizophrenia spectrum disorders. Multiple systematic reviews and meta-analyses have evaluated their effectiveness, but findings vary. This umbrella review of systematic reviews and meta-analyses aims to synthesise the highest-level evidence by conducting an umbrella review of systematic reviews and meta-analyses comparing LAIs with OAPs in patients with early psychosis.

**Methods:**

A literature search was conducted to identify systematic reviews and meta-analyses of LAIs versus OAPs. Eligible studies included reviews published up to December 2025 and reported clinical outcomes including symptom change, relapse, hospitalisation, treatment discontinuation, and adverse effects. Findings were synthesised narratively with focus on consistency across reviews and strength of evidence.

**Results:**

Eleven eligible reviews (seven systematic reviews and four meta-analyses) wereidentified, together covering 58 unique primary studies with sample sizes ranging from 220 to 14,313 participants. Across reviews, LAIs generally demonstrated a positive impact on symptomatic outcomes in early psychosis. Most reviews noted greater reduction in discontinuation due to non-adherence or inefficacy with LAIs versus OAPs; however, meta-analytic results did not consistently show significant differences in overall discontinuation or relapse prevention. Evidence regarding relapse was heterogeneous, with some reviews suggesting benefit and others showing inconclusive results. Reports of metabolic side effects were similar between formulations, with some indication of lower extrapyramidal symptoms with LAIs.

**Conclusion:**

Umbrella-level evidence suggests that LAIs may offer advantages over oral antipsychotics in certain outcomes, particularly related to adherence, but there is no clear consensus that LAIs uniformly outperform OAPs across all clinical endpoints in early psychosis. These findings highlight the need for further high-quality pragmatic research to refine clinical guidance on LAI use.

